# From Wikipedia to AI: Measuring 25 years of synthesis of human genetics research in the public-facing information ecosystem

**DOI:** 10.64898/2026.07.27.741055

**Published:** 2026-08-04

**Authors:** Alex Diaz-Papkovich, Abigail Kuntzleman, Sophia C. Davis, Sohini Ramachandran

## Abstract

Genetics research frequently intersects with ethnicity, nationality, and race, making it uniquely vulnerable to misrepresentation. Yet, 25 years after the initial sequencing of the human genome, there is little understanding of how human genetics research exists in the public-facing information ecosystem. We analyze 3,050,422 historical revisions from 6,738 Wikipedia pages about ethnicity, nationality, and race spanning 25 years. We find genetics terminology is present in 14.8% of these pages (55.5% in the top 1,000 pages) and in 67.8% of pages about nationalities, suggesting research is synthesized to present a biological element to ethnicity and nationality. We also find that 10.1% of 56,908 discussions from these pages contain genetics terminology. We further analyze responses from three popular chatbots queried about nationalities and find that they commonly reference both genetics and Wikipedia. Lastly, we analyze 133 pages from Grokipedia, an AI-generated encyclopedia, and find it mentions genetics more frequently than Wikipedia and hallucinates or misrepresents human genetics research.

## Full Text Availability

The license terms selected by the author(s) for this preprint version do not permit archiving in PMC. The full text is available from the preprint server.

